# Section on Ophthalmology—College of Physicians of Philadelphia

**Published:** 1900-12

**Authors:** 


### SECTION ON OPHTHALMOLOGY—COLLEGE OF PHYSICIANS OF PHILADELPHIA.

Meeting October 16, 1900. Dr. George C. Harlan, chairman, in the chair.

Dr. William Thomson exhibited two men recently operated upon at Wills hospital for removal of metallic foreign bodies by the small magnet from the posterior portion of the eyeball. In one case, a piece of iron, 8 mm. by 1.5 mm., penetrated the inner side of cornea near the horizontal meridian, and was located by the X-rays embedded at a point 5 mm. below and 3 mm. to the temporal side of the macula. The body was removed through a scleral incision midway between external and internal recti. Very little reaction followed. In the second case a chip of steel entered the cornea, 2 mm. above and 2 mm. to the outer side of the center wounding iris and lens, and embedding itself, as shown by the radiographs, 2 mm. above and 6 mm. to nasal side of macula. The partially transparent lens was extracted through the usual incision, an iridectomy performed, and the point of the magnet passed to the supposed situation of the metal. Upon its removal, a piece of steel 1.5 mm. by 1 mm. was found attached to the magnet point. Three weeks after operation the eyeball was markedly injected, with lessened tension.

Dr. George C. Harlan showed an Italian girl, fourteen years of age, presenting the history and the ophthalmoscopic appearances of embolism of the central retinal artery, except that the arteries contain blood and pulsate freely on pressure. The patient has menstruated regularly for two years, and seems to be in perfect health. Sight was suddenly lost in the right eye on October 7th, and when she came to the Pennsylvania Hospital two days later, there was then a faint perception of light revealed by the flash of the mirror, which has since given place to absolute blindness. The whole fundus, except the extreme periphery, presented a grayish-white color, most dense about the macula and disk, and shading off gradually to nearly normal red at the periphery. The red spot at the macula was darker than the typical "cherry-red." The vessels were obscured in places by the retinal opacity, but reappeared. The arteries were normal in caliber and pulsated freely under pressure. The veins were slightly engorged. There were no hemorrhages. On October 16th the arteries are somewhat narrowed, but still pulsate under moderate pressure, the veins less engorged and of lighter red, and the red spot at macula also lighter, while two bright-red punctate hemorrhages have appeared in the macular region. There is no marked change in the general color of the fundus, but the disk is decidedly pale. Considering the full arteries and distended veins, the most probable diagnosis seemed at first to be venous thrombosis, but the subsequent narrowing of all the vessels and the absence of considerable extravasations do not encourage this view. Incomplete embolism is more probable, but the pathology is very obscure. The urine and blood are normal. Dr. de Schweinitz suggested hemorrhage in the nerve-sheath as a possible cause.

Dr. Risley exhibited a man from the Wills Hospital, fifty-nine years of age, upon whom he had performed simple extraction of cataract in each eye. The case was presented because of the unusually favorable results which had been reached. Vision was $\frac{6}{V}$

in each eye, and the man enjoyed perfect binocular vision both for distance and in reading; the pupils were central and round, reacted normally to change of light and shade, and there were no adhesions in either eye, either to the capsule or the cornea in the region of the wound. The cicatrix in the line of the incision was visible only on careful inspection, and was then found to lie in the clear corneal limbus. Dr. Risley said that he had never before been able to secure in both eyes of the same patient an ideal result. While this was not unusual in one eye, some error in technic or accident during convalescence had always marred to some extent the result in one eye. In conversation with his colleagues he had been led to believe that this had been their experience also.

Dr. William M. Sweet reported a case of a piece of glass in the ciliary body located by the Roentgen rays and its removal with forceps. The injury occurred to an engineer from the explosion, on March 1st, of a locomotive oil-glass, a fragment penetrating the cornea in the lower outer quadrant near the sclera, and passing through iris and periphery of lens. When first seen, three months after the accident, the lens had been absorbed, but haziness of the vitreous prevented a view of the interior of the globe. Radiographs indicated that a body, casting a fainter shadow than is usual with metallic substances, was situated close to the ciliary body at its lower temporal portion. On October 6th an iridectomy was performed at the Jefferson Hospital through an incision at the lower outer corneo-scleral junction, and a pair of iris forceps passed to the spot indicated by the radiographs. After a number of attempts, a mass of exudate containing the piece of glass was drawn into the anterior chamber and removed. Warm saline solution was injected into the globe to replace the vitreous lost. The glass was 5 mm. long, 3 mm. wide, and 1.5 mm. thick. Very little reaction followed the operation, the corneal wound healing on the third day and the man being discharged on the eighth day. The vitreous is still hazy, but the eye is quiet, with prospect of a serviceable ball and possibly some sight.

*Concerning Traumatic Palsies of the Ocular Muscles, with Cases.*—Dr. C. E. de Schweinitz, after a brief reference to the literature of traumatic paralysis of the ocular muscles, reported the following cases: Paralysis of the left inferior rectus from a knife thrust, advancement of the severed tendon and tenotomy of the superior rectus, cure; division of the pulley of the superior oblique during an operation for abscess of the orbit caused by suppurating ethmoiditis, spontaneous disappearance of the diplopia, restoration of the normal rotations of the eye; injury of the superior oblique and superior rectus during an operation for the removal of a tumor from the orbit, the patient still under treatment; paresis of the right inferior rectus following a fall on the right side of the head, spontaneous recovery; paralysis of the left superior rectus and left levator palpebræ following a fall upon the left side of the head and forehead, spontaneous disappearance of the ptosis, persistence of the paralysis of the left superior rectus, binocular single vision secured by tenotomy of the left inferior rectus; paresis of the right superior oblique following a fall on the right supra-orbital region, later paresis of accommodation, complete recovery; paresis of the left inferior rectus following a fall on the head succeeded by concussion of the brain, recovery; paralysis of the left superior oblique following a fall on the head succeeded by severe concussion of the brain, recovery.

Of the five cases of palsy not produced by direct traumatism Dr. de Schweinitz attributed two of them to a basal lesion, one almost certainly being associated with fracture, one to a nuclear lesion in the anterior portion of the third nerve nucleus, and the other possibly to a nuclear lesion. In one case the evidences of injury were so slight, with the exception of the paralysis of the inferior rectus, that no satisfactory localization of the lesion could be made.

*Discussion*—Dr. S. D. Risley recalled the history of a case presented to the Section last year, of a man who had been thrown from his buggy and remained unconscious for two days, besides suffering from a large lacerated wound of the scalp above the right frontal region. When seen three months later there was a nearly complete paralysis of all of the ocular muscles on the right side supplied by the third nerve except the levator. He did not know the later history, but thought it obviously belonged to the last group of cases. He presented the history of two cases which properly belonged to the first group. A lad had received a thrust from the ferrule of an umbrella, which detached the whole or a large portion of the tendon of the internal rectus and lacerated extensively the conjunctiva and capsule from the upper and inner quadrant of the eyeball, pushing it backward. When seen in consultation with Dr. John T. Carpenter, Jr., these tissues had fastened themselves to the sclera in their abnormal position, and diplopia existed. The tendon of the internus and the torn tissues were separated from their faulty attachment and brought forward by Dr. Carpenter, with the result that the diplopia disappeared. In the second case the tendon of the inferior rectus had been severed from the ball with considerable laceration of the capsule to the temporal side, causing vertical diplopia, violent attacks of headache, and inability to use the eyes. There was marked rotation of the vertical meridian of the cornea. The tendon of the internus was brought forward, with the result of relieving the vertical diplopia except during fatigue, or when looking downward and to the left, when there would first be considerable confusion of vision, then a recurrence of the diplopia with nausea and occipital pain. The capsule and overlying tissues at the temporal side of the inferior rectus were then detached and brought forward, and the diplopia and asthenopia quickly disappeared. Dr. B. Alexander Randall referred to a case of paralysis of the inferior rectus, decreasing when he first saw the man, in which complete recovery followed absorptive treatment. In a child with paralysis of the superior rectus and levator muscles, from penetration of a cow's horn, perfect success followed picking up the rectus muscle and stitching it in place, but the result of operation upon the levator muscle was not so good.

WILLIAM M. SWEET,
*Clerk of Section.*

